# Neurogenic Orthostatic Hypotension in Parkinson Disease—A Narrative Review of Diagnosis and Management

**DOI:** 10.3390/jcm14020630

**Published:** 2025-01-19

**Authors:** Cristina Grosu, Otilia Noea, Alexandra Maștaleru, Emilian Bogdan Ignat, Maria Magdalena Leon

**Affiliations:** 1 Department of Neurology, “Grigore T. Popa” University of Medicine and Pharmacy, 700115 Iași, Romania; cristina.grosu@umfiasi.ro (C.G.); emilian.ignat@umfiasi.ro (E.B.I.); 2 Department of Neurology, Rehabilitation Hospital, 700661 Iași, Romania; otilian18@yahoo.co.uk; 3 Department of Medical Specialities I, “Grigore T. Popa” University of Medicine and Pharmacy, 700115 Iași, Romania; maria.leon@umfiasi.ro

**Keywords:** Parkinson disease, neurogenic orthostatic hypotension

## Abstract

**Background:** Neurogenic orthostatic hypotension (NOH) is a significant non-motor manifestation of Parkinson’s disease (PD), that substantially affects patient disability and has a powerful impact on the quality of life of PD patients, while also contributing to increased healthcare costs. This narrative review aims to summarize key insights into the diagnosis and management of NOH in individuals with PD. **Methods:** For diagnosing NOH, a recently introduced and valuable metric is the ΔHr/ΔSBP index. Additional tools, such as autonomic reflex testing and various blood tests, also can be used to help distinguish orthostatic hypotension (OH) from NOH. **Results:** Treatment strategies for NOH involve both non-pharmacological and pharmacological approaches. As NOH frequently coexists with other abnormal blood pressure patterns (supine hypertension, nocturnal hypertension, and non-dipping hypertension), its treatment can be a challenge for the clinician. Droxidopa and midodrine are the primary pharmacological agents for NOH, though emerging therapies, such as norepinephrine transporter inhibitors, are being investigated. **Conclusions:** Despite these advancements, further research is needed to better understand the underlying pathophysiology of NOH, enabling more tailored and effective treatment options for individuals with PD.

## 1. Introduction

Parkinson’s disease (PD) is a progressive neurodegenerative disorder primarily characterized by the loss of dopaminergic neurons in the substantia nigra. It presents with a combination of motor and non-motor symptoms. Key motor manifestations, which are central to diagnosis, include bradykinesia, rigidity, and tremor. Non-motor symptoms encompass hyposmia, sleep disturbances, cognitive impairment, depression, psychosis, dementia, and autonomic dysfunction. Autonomic manifestations in PD consist of gastrointestinal dysfunction such as delayed gastric emptying, constipation, and dysphagia; urinary symptoms like urgency of micturition; erectile dysfunction and cardiovascular disorders, including neurogenic orthostatic hypotension, neurogenic supine hypertension, and nocturnal hypertension [1,2].

Orthostatic hypotension (OH) affects approximately 30% of individuals in the PD in population, and is recognized as a diagnostic marker of prodromal PD, according to the Movement Disorder Society research criteria [3]. OH significantly contributes to functional disability in people with PD by increasing the risk of falls, and it is associated with higher healthcare resource utilization, independent of age, disease duration, or motor symptom severity. A study involving 317 PD patients found that the presence of OH led to a 285% increase in hospitalization days and a 152% rise in emergency room visits [4,5]. Additionally, OH has also been linked to cognitive impairment, faster disease progression, and increased mortality [6]. This review aims to provide a comprehensive overview of the diagnosis and management of neurogenic orthostatic hypotension (NOH) in individuals with PD.

## 2. Diagnosis of NOH

Neurogenic orthostatic hypotension (NOH) is defined as a sustained decrease in systolic blood pressure (SBP) of at least 20 mmHg, diastolic blood pressure (DBP) of at least 10 mmHg, or both within three minutes of standing or being tilted of at least 60° on a tilt table. Ideally, the blood pressure (BP) is measured after the patient has been lying down for five minutes, then again at one and three minutes after standing. It is important to note that some individuals experience delayed OH, where the drop in BP occurs after more than three minutes of standing, and this can progress to classic OH [7]. An increase in heart rate (HR) of less than 15 beats per minute (bpm) is considered highly suggestive of NOH [3,4,5,6,7,8]. In a multicenter study of 402 patients, 378 of whom had degenerative synucleopathies, Kaufmann and colleagues, found that a ΔHr/ΔSBP ratio of less than 0.49 had a sensitivity of 91.3% and a specificity of 88.4% in distinguishing NOH from non-neurogenic OH. In contrast, an HR increase of less than 17 bpm demonstrated lower sensitivity (79%) and similar specificity (87%). In a validation study, the ΔHr/ΔSBP ratio of less than 0.49 showed a sensitivity of 91% but a reduced specificity of 59%, compared to the study of Kaufman et al. ΔHR less than 15 bpm had 84% sensitivity and 50% specificity. These studies suggest that a ΔHr/ΔSBP ratio less than 0.49 may help identify patients who require further autonomic function testing in order to establish a definitive diagnosis of NOH [9].

The relationship between motor symptom laterality (where symptoms predominantly affect either the left or right side) in PD and the occurrence or severity of NOH remains an area with limited and inconclusive research, and also, its direct influence on autonomic dysfunction, including NOH, is not well established. Some studies suggest that left-sided motor symptom predominance (LPD) in PD may be associated with greater motor and autonomic dysfunction, including cardiovascular irregularities, due to more significant right hemisphere involvement and, therefore, overall higher burden. Interestingly, despite these impairments, LPD patients reported better QoL scores than right-sided motor symptom predominance (RPD) patients, possibly due to the dominant use of the right hand in daily activities or decreased awareness of motor issues due to right hemisphere involvement [10]. The right hemisphere has been implicated in autonomic regulation, particularly in controlling sympathetic cardiovascular responses, and this could theoretically mean that patients with LPD might experience more pronounced autonomic issues, such as NOH, due to right-hemisphere degeneration [11]. However, this association has not been consistently demonstrated across studies, and more focused research is needed to clarify whether motor laterality significantly affects the risk or severity of NOH in PD. Additionally, autonomic dysfunction in PD, including NOH, is more commonly associated with disease progression and widespread neurodegeneration rather than lateralized motor symptoms. Studies on PD subtypes have primarily focused on cognitive impairment, mood disorders, and motor complications in relation to motor laterality, while research directly linking laterality to autonomic symptoms like NOH remains scarce [12].

Ambulatory blood pressure measurement (ABPM) is recommended as it can detect OH, as well as other abnormal hypertensive patterns frequently seen in PD patients, such as neurogenic supine hypertension (nSH), nocturnal hypertension, and non-dipping [1]. Therefore, it represents an important step toward a tailored therapeutic approach. The accuracy of the ABPM interpretation improves when patients maintain a diary documenting medication intake times, physical activities, meals, and instances of getting out of bed at night [13].

Wearable devices have emerged as valuable tools for the continuous monitoring of NOH in patients with PD, offering a more dynamic and real-world assessment of BP fluctuations than traditional clinical methods. Devices such as ambulatory BP monitors, smartwatches, and biosensors can track BP and HR variability throughout daily activities, providing insights into intermittent and unpredictable episodes of OH that may not be captured during routine clinical evaluations [14]. Continuous monitoring allows clinicians to detect symptomatic and asymptomatic episodes of NOH, assess the frequency and severity of BP drops, and understand the impact of posture changes on cardiovascular stability. These real-time data are crucial for developing more personalized treatment plans and reducing the risk of falls and injuries associated with hypotensive episodes in PD patients. Recent advancements in wearable technology have led to the development of non-invasive, cuffless BP monitors that use pulse transit time (PTT) and photoplethysmography (PPG) to estimate BP continuously. Devices like smartwatches equipped with PPG sensors and accelerometers can detect posture changes and provide BP trends over time, offering a convenient and less intrusive method for long-term monitoring [15]. Additionally, wearable biosensors integrated with smartphone applications allow patients to share their data with healthcare providers in real-time, facilitating timely adjustments in therapy. These technologies enhance patient engagement in disease management and enable proactive intervention, potentially improving outcomes for PD patients experiencing NOH [16].

However, despite the promising role of wearable devices in managing NOH, several challenges remain. The accuracy and reliability of wearable blood pressure monitors can be influenced by factors such as body movement, skin tone, and device calibration, which may limit their clinical utility without standardized validation protocols. Furthermore, integrating wearable data into clinical workflows requires secure data handling and effective interpretation to inform treatment decisions. Ongoing research is needed to validate the efficacy of these devices in PD-specific populations and to establish standardized guidelines for their clinical use [17]. Nonetheless, wearable technology represents a significant advancement in the continuous and personalized monitoring of NOH, potentially improving safety and quality of life for individuals with PD.

Autonomic reflex testing includes evaluating HR and BP response to the Valsalva maneuver (which assesses adrenergic function), HR response to deep breathing (to test cardiac parasympathetic function), and head-up tilt testing (HUT). Additionally, sudomotor function assessments, such as the quantitative sudomotor axon reflex test, may also be performed [18]. The Valsalva maneuver assesses autonomic and cardiovascular responses by having the patient perform forced exhalation against a closed airway for about 15 s. It involves four phases, highlighting changes in BP and HR during strain and release. The tilt table test evaluates blood pressure and heart rate responses to positional changes. The patient is tilted to an upright position at 60–80 degrees for 10 to 20 min, while monitoring for significant BP drops or symptoms like dizziness, which confirm OH. Table 1 describes the comparative diagnostic tests for NOH in PD.

Patients with OH should undergo a series of tests to differentiate between OH and NOH. These include an electrocardiogram (ECG) to rule out possible arrythmias that could prevent a compensatory HR increase during orthostasis, a complete blood count to exclude anemia or infection, and a basic metabolic panel, including electrolytes, bicarbonate, blood urea nitrogen (BUN), creatinine, fasting glucose, and HbA1c. Testing thyroid-stimulating hormone (TSH) and vitamin B12 levels is also essential, as thyroid dysfunction or vitamin B12 deficiency can impair autonomic function [3].

Due to the fact that the accurate diagnosis of NOH in PD patients can be challenging mostly to the overlap of symptoms with other PD-related autonomic dysfunctions (such as supine hypertension, postprandial hypotension, and medication-induced hypotension), a potential risk arises for both the overdiagnosis and misdiagnosis of NOH, leading to inappropriate or delayed treatment strategies that could compromise patient outcomes [19,20]. The diagnostic complexity is further compounded by the episodic nature of NOH and variability in clinical presentation, which can make it difficult to distinguish from non-neurogenic forms of OH or transient BP fluctuations. Standard diagnostic tools like the active stand test or tilt-table testing might not always capture intermittent BP drops, especially in early or fluctuating stages of PD. Additionally, the presence of supine hypertension, commonly coexisting with NOH in PD, can obscure clinical assessment and treatment decisions, as interventions for one condition may exacerbate the other [21]. This delicate balance often results in a cautious or even inaccurate diagnostic approach, raising concerns about both under-and over-recognition of NOH in clinical practice.

Clinically, the misdiagnosis of NOH can lead to inappropriate management, such as the unnecessary use of volume expanders or vasoconstrictors, which may worsen coexisting cardiovascular issues or supine hypertension. On the other hand, failure to diagnose NOH can increase the risk of falls, fractures, and reduced quality of life due to unmanaged hypotensive episodes. This underscores the importance of comprehensive autonomic testing, careful medication review, and longitudinal BP monitoring in PD patients to differentiate NOH from other autonomic dysfunctions. Improving diagnostic accuracy through the integration of wearable BP monitors and refining diagnostic criteria may help mitigate misdiagnosis and enhance patient care [22,23].

## 3. The Role of Medication in NOH Management of PD Patients

Conducting a medication review is an essential aspect of history taking, as many drugs that can either induce OH or worsen its symptoms. The cardiovascular medication most frequently linked to OH include diuretics (due to volume depletion), alpha-blockers (which reduce vascular resistance), beta-blockers (which have a chronotropic and inotropic effect that minimize compensatory heart rate response to orthostasis), nitrates, and calcium channel blockers. For OH patients who also have benign prostatic hyperplasia, prescribing more uroselective alfa-blockers (e.g., silodosin) is recommended. Additionally, drugs like carvedilol, which block alpha and beta receptors, should be avoided [24].

Among psychoactive medication, tricyclic antidepressants carry a significant risk of OH due to their ability to reduce vascular resistance by blocking alpha receptors. In contrast, OH is less frequently associated with selective serotonin reuptake inhibitors (SSRIs) and serotonin-norepinephrine reuptake inhibitors (SNRIs). Antipsychotics, which also block alpha1-adrenergic receptors, like clozapine, quetiapine, and chlorpromazine, have the highest risk of OH [24]. These considerations are particularly relevant for PD patients, as depression and psychosis are common non-motor symptoms in the course of this neurodegenerative disorder [25].

Antiparkinsonian drugs have also been associated with OH. Although there is controversy surrounding the definite mechanism through which levodopa causes a reduction in the BP, it is thought to involve vasodilation and baroreflex dysfunction [26]. A systematic review of randomized controlled trials found no increased risk of OH with monoamine oxidase B inhibitors (IMAO-Bs) or dopamine agonists compared with placebo. Nevertheless, it worth noting that the participants in the included studies, were younger than typical PD populations, and OH was not specifically reported in most trials [27]. A prospective study involving 99 PD patients, also did not find a statistically significant difference in BP drops after orthostasis between patients taking levodopa and those not on medication [28].

Cani et al. examined the impact of levodopa on BP and HR in 164 patients with parkinsonism undergoing chronic levodopa treatment. They observed a decrease in BP in both supine and orthostatic positions following the subacute oral administration of levodopa. Their findings also revealed that patients with autonomic dysfunction presented with a higher risk of developing levodopa-induced OH [29]. Additionally, a study of 490 PD patients, more than half experienced acute OH post-levodopa, with the effect most commonly occurring within the first hour after administration. A better motor responsiveness to levodopa was associated with a higher risk for acute OH post levodopa (AOHPL) [26].

The administration route of levodopa also seems to influence its impact on OH. In a pilot study of PD patients, those receiving levodopa–carbidopa intestinal gel, experienced significant relief from OH-related symptoms compared to those on optimized medical treatment. However, other studies did not find a significant association between levodopa-carbidopa intestinal gel administration and the presence of OH symptoms [30].

## 4. Symptoms

The most common symptoms of OH include lightheadedness, dizziness, and blurred vision, which are caused by reduced brain perfusion [31]. Syncope associated with NOH is not typically accompanied by other autonomic symptoms such as diaphoresis, nausea, or abdominal discomfort [14]. Patients may also report neck and shoulder pain (referred to as “coat-hanger pain”), orthostatic dyspnea, chest pain, generalized weakness, fatigue, and nausea. Notably, the immobility that occurs in PD patients and mimics the “levodopa off state” can sometimes be caused by OH [32].

A significant number of patients with NOH are asymptomatic. Although a precise mechanism underlying “hypotension unawareness” remains unclear, global cerebral hypoperfusion has been suggested as a possible explanation. A prospective study involving 89 patients found no correlation between systolic blood pressure (SBP) drops and reported symptoms, with a large percentage of patients remaining asymptomatic despite substantial SBP decreases [33].

According to a survey that sought to characterize the symptoms of NOH, most patients tend to minimize their symptoms and are reluctant to discuss them with healthcare providers. Asymptomatic NOH is particularly important because it has also been associated with a higher risk of falls and cognitive decline [34]. Furthermore, in another study of 121 patients, asymptomatic OH was found to cause similar impairment in activities of daily living (ADL), instrumental activities of daily living (iADL), and ambulatory capacity measures (ACM) comparable to those caused by symptomatic OH [5].

## 5. Physiopathology

In healthy individuals, standing up triggers an initial drop in BP, which is detected by baroreceptors. This triggers a compensatory response involving an increase in HR and vasoconstriction, mediated by norepinephrine released from postganglionic sympathetic efferent nerves [35]. In PD, degeneration of dopaminergic neurons in the substantia nigra extends to involve the autonomic nervous system, particularly the sympathetic and parasympathetic pathways. Key structures affected include the intermediolateral cell column of the spinal cord and peripheral sympathetic ganglia, leading to reduced norepinephrine release. This impairs vasoconstriction and the baroreflex response, which is crucial for maintaining blood pressure during positional changes. Additionally, cardiac sympathetic denervation, confirmed via reduced myocardial uptake of radioactive tracers like ^123^I-MIBG, contributes to decreased heart rate variability and diminished ability to compensate for sudden gravitational shifts when standing. These mechanisms result in a significant drop in BP upon standing, causing dizziness, syncope, and other symptoms characteristic of OH [36].

In Lewy-body disorders (LBD), which encompasses dementia with Lewy bodies and overlaps with PD, NOH primarily results from the degeneration of peripheral postganglionic sympathetic neurons and accumulation of alpha-synuclein inclusions, particularly in the autonomic nervous system. This includes cardiac, leading to a reduced tachycardia response, and extra-cardiac neurons, causing decreased peripheral vasoconstriction. As in PD, alpha-synuclein pathology in LBD disrupts norepinephrine release and compromises baroreflex function, resulting in reduced vascular tone and a diminished cardiac output response to orthostatic stress. However, in LBD, the autonomic dysfunction may be more pronounced early in the disease course, reflecting the broader distribution of Lewy bodies across the brainstem, hypothalamus, and autonomic ganglia [37].

In multiple system atrophy (MSA), a neurodegenerative disorder characterized by widespread autonomic failure, OH is a hallmark feature. Unlike PD, where cardiac sympathetic denervation is variable, MSA demonstrates consistent pre- and postganglionic sympathetic nervous system involvement, mainly caused by degeneration of the intermediolateral columns of the spinal cord, brainstem nuclei, and peripheral autonomic neurons. Consequently, patients experience severe impairment in baroreceptor reflex arcs and vasomotor control, leading to inadequate vascular resistance and blood accumulation in the lower extremities upon standing. The severity of OH in MSA correlates with disease progression and is often refractory to conventional treatments, posing significant management challenges [38].

Sleep disturbances are also linked to NOH, particularly in the context of idiopathic REM sleep behavior disorder (iRBD), which is often found to precede some alpha-synucleinopathies such as PD, MSA, or LBD. In iRBD, neurodegeneration affects the brainstem nuclei, such as the locus coeruleus and dorsal motor nucleus of the vagus, areas that modulate sympathetic and parasympathetic activity, and their dysfunction contributes to both NOH and disrupted REM sleep. The overlap between autonomic and sleep dysregulation can lead to nocturnal blood pressure variability, impaired cardiovascular reflexes, and reduced baroreflex sensitivity, exacerbating NOH. Sleep fragmentation further worsens autonomic instability by disrupting the restorative phases of sleep necessary for homeostatic control, and therefore, iRBD patients with emerging autonomic dysfunction often experience morning hypotension, daytime fatigue, and a higher risk of falls and syncope [39].

Another interesting subject that emerges nowadays is the relationship between iRBD, NOH, and positive neurological symptoms such as REM sleep behavior (acting out dreams) that can be explored through frameworks like the Defensive Activation Theory (DAT) and Active Inference Theory (AIT). DAT suggests that dream enactment behaviors (like hallucinations or involuntary movements) in iRBD arise from the brain’s failure to suppress motor activity during REM sleep due to degeneration of inhibitory pathways in the brainstem, which regulate autonomic function and are damaged in NOH and iRBD. AIT suggests that the brain constantly updates its internal models based on sensory input to minimize prediction errors and extends this by proposing that positive neurological symptoms (including REM enactment and autonomic instability) reflect errors in the brain’s predictive coding mechanisms, therefore misinterpreting sensory inputs (like low BP signals) and generates maladaptive autonomic or motor responses. In iRBD, this could manifest as dream-related motor activation and impaired baroreflex responses, linking sleep disturbances to autonomic failure. Also, the critical role of dopamine in facilitating neuroplasticity, enabling the brain to adapt to damage, is to be investigated further on as dopamine receptor agonists may exacerbate hallucinations by overstimulating this adaptive mechanism, while antagonists suppress it, potentially hindering the brain’s compensatory responses. This theoretical perspective of both DAT and AIT suggests that positive symptoms may not merely signal disease progression but could indicate the brain’s active efforts to maintain homeostasis and offer a new understanding of how neurodegeneration in alpha-synucleinopathies affects all together sleep, motor, and autonomic dysfunctions, highlighting early diagnostic and therapeutic opportunities [40].

## 6. Treatment of NOH

Treatment of NOH comprises pharmacological and non-pharmacological measures. The purpose of treatment is not to normalize standing BP, but to improve symptoms, the quality of life, reduce the risk of falls, and increase patients’ independence in daily activities.

### 6.1. Non-Pharmacological Measures

The most important measure is patient education. The patient should be instructed to recognize presyncopal symptoms to avoid triggers such as hot baths, alcohol, and large meal intakes, and sudden transitions from a supine position to standing. Sleeping with the head of the bead elevated reduces nocturnal diuresis and natriuresis and also lowers supine hypertension [33]. Patients should also avoid Valsalva-like maneuvers (e.g., straining during defecation), making it important to promptly manage constipation, a common non-motor symptom in PD patients. Drinking 500 mL of water within 5 min, particularly in the morning, can also be beneficial.

Physical counter maneuvers can be an efficient method to prevent OH symptoms—for example, crossing legs while standing, bending forward, squatting, and contracting the abdominal and leg muscles. Squatting is especially useful in cases of imminent syncope as it quickly enhances venous return, thus increasing cardiac output [41]. However, it should be taken into consideration that lower extremity counter maneuvers may be unsafe for PD patients, who are already prone to falls than other populations. RULE (resisted upper limb exercise) can complement lower limb counter-maneuvers, offering an alternative mechanism to improve OH.

Exercise plays an important role in OH treatment, as it prevents deconditioning, which can worsen orthostatic intolerance by reducing left ventricular chamber function [33]. However, one should keep in mind the higher risk of exercise-induced hypotension and post-exercise OH in PD patients [42]. Therefore, activities such as a recumbent stationary bicycle or rowing machine may be more suitable for these patients [43]. A randomized clinical trial involving PD patients demonstrated a significant improvement in systolic BP response to orthostasis after a 12-week progressive resistance training program [44]. Another study indicated that four weeks of treadmill training positively affected baroreflex control of BP [43]. Research on PD patients with OH also found that RULE reduced OH and increased systemic BP. Unlike aerobic exercise involving the lower limbs, which can worsen OH due to increased blood pooling in the lower body, RULE does not carry this risk and is, therefore, more suitable for PD patients. However, further studies are needed to investigate the impact of exercise on autonomic dysfunction in PD patients, including which types of exercise are the most beneficial, the optimal duration of training, and the potential interactions with medication [45].

Compression stockings can enhance venous return and thus increase BP. Notwithstanding, they may be difficult for PD patients to use due to limited mobility. In such cases, abdominal binders represent a reliable alternative. A study on PD patients showed that elastic abdominal binders led to a significant reduction in OH symptoms when elastic without causing a substantial change in supine BP [46]. Another study supported the use of abdominal binders with a pressure of 20 ± 2 mmHg and emphasized that they should not be removed when the patient stands, as that can worsen OH symptoms and negatively impact cerebral hemodynamics [47].

### 6.2. Pharmacological Measures

Pharmacological treatment plays an essential role in managing orthostatic hypotension in Parkinson’s disease patients. These treatments aim to enhance blood pressure regulation and improve symptoms by targeting various mechanisms within the autonomic nervous system. Commonly used medications include fludrocortisone and midodrine, which help increase blood volume and promote vasoconstriction to maintain blood pressure when standing. Another option is the use of droxidopa, a norepinephrine precursor, that supports sympathetic activity and BP regulation or other options, such as sildenafil and certain serotonin reuptake inhibitors. Treatment should be personalized in order to ensure the best therapeutic outcomes.

**Droxidopa** is a synthetic amino acid that is converted into norepinephrine by dopa-decarboxilaze, the same enzyme that facilitates the conversion of levodopa into dopamine. It causes peripheral arterial and venous vasoconstriction, thus raising standing blood pressure. It was approved by the FDA after three RCTs showed a significant improvement of NOH symptoms, a good impact of symptoms on activities of daily life, and also a statistically significant increase in standing BP when compared to placebo [1]. It should also be noted that although DOPA decarboxylase inhibitors (DDCIs) were used in the majority of patients, they did not have a negative impact on droxidopa efficacy. This is important as PD patients treated with levodopa will always be prescribed a combination of levodopa and DDCI; DDCI blocks the conversion of droxidopa to norepinephrine [48].

Droxidopa reaches peak plasma concentration within approximately 3 h after oral administration. Although in clinical studies, the drug is administered in doses of 100–600 mg, three times daily, it is recommended that the optimal dosage be calculated through a titration procedure, and also administration should be individualized, taking into consideration the daily activities of each patient. Unfortunately, not all patients respond to droxidopa. A small observational study on 20 patients with NOH showed that the ones with supine venous plasma levels of norepinephrine below 220 pg/mL (which suggests postganglionic sympathetic denervation) have a better response to droxidopa [44].

**Midodrine**, an oral pro-drug that is converted into desglydomidodrine and acts as an alpha-1 adrenoreceptor agonist, raising BP by causing vasoconstriction of arterioles and veins. This medication is short-acting, with its maximum BP effect occurring about one hour after administration. Due to the risk of supine hypertension, midodrine should not be taken within 5 h of bedtime [1]. The recommended dosage is 3.5–15 mg per dose, taken 1–3 times per day [3]. A meta-analysis by Parsaik and colleagues, which included seven trials, found that midodrine could increase standing SBP and improve overall symptoms; however, it did not show a significant positive impact on the change from supine to standing SBP. The analysis also revealed a high incidence of side effects, with pooled risk ratios of 6.38 for supine hypertension, 10.53 for piloerection, 6.45 for scalp pruritus, 8.28 for scalp paresthesia, and 5.85 for urinary retention [49].

A retrospective study comparing patients treated with droxidopa or midodrine for OH found that treatment persistence was significantly higher in the droxidopa group compared to the midodrine cohort. Patients on droxidopa monotherapy were 16% more likely to continue treatment than those on midodrine (*p* < 0.001). Additionally, PD patients were more likely to remain on therapy [50]. A meta-analysis including six randomized trials that compared droxidopa and midodrine with the placebo for the treatment of NOH, showed a greater mean increase in standing SBP from baseline in the midodrine group (17 mmHg) compared to the droxidopa group (6.2 mmHg). While midodrine was associated with a significantly higher risk of supine hypertension compared to placebo, this risk was not observed with droxidopa [51].

**Fludrocortisone**, a synthetic mineralocorticoid which increases sodium retention, is commonly used off-label for OH treatment. It expands plasma volume and promotes vascular resistance. However, it has been associated with multiple adverse effects, such as supine hypertension, edema, congestive heart failure, hyperkaliemia, and headache. A recent Cochrane review of several RCTs found limited evidence on the efficacy of fludrocortisone, with the quality of evidence being low/very low, especially concerning its long-term use, as most RCTs had short duration and small sample sizes [52].

**Pyridostigmine** is an acetilcholinesterase inhibitor that increases cholinergic transmission in the autonomic ganglia. Its main advantage consists of the fact that it favors sympathetic activation only when the patient is standing, thus, not increasing the risk of supine hypertension [32]. Pyridostigmine raises standing systolic BP on average with 4 mmHg and its usual dosage is 30–60 mg, administered three times per day. The main side effects are due to cholinergic activation and include salivation, urinary urgency, abdominal cramps, and diarrhea.

**NET inhibitors** are another class of drugs which showed promising results in the treatment of NOH. While atomoxetine is a short-acting agent, ampreloxetine is a newer medication with the advantage of a longer plasma half-life (30–40 h). Urinary tract infection represented the most frequent adverse effect of ampreloxetine (23.8%), followed by hypertension and headache. Although a small phase 2 trial showed promising results for ampreloxetine in treating NOH, a press release from the company reported the phase 3 study as a failure [53]. A recent meta-analysis examining atomoxetine for NOH in patients with alfa-synucleopathies concluded that it could safely be used as a short-term treatment for NOH, potentially more effective in cases involving central autonomic dysfunction, such as multiple system atrophy (MSA). However, further studies are needed to evaluate its efficacy in PD patients [54].

Interestingly, a recent study conducted by Okamoto and colleagues discovered that combining atomoxetine and pyridostigmine had a significant synergistic pressor effect in patients with severe autonomic failure and NOH, whereas each drug alone did not improve either BP or orthostatic tolerance. Nonetheless, additional research on the safety and long-term efficacy of this combined treatment approach is necessary [55].

**Domperidone**, a D2 receptor antagonist, has been suggested as a treatment option for alleviating OH caused by dopaminergic medications in PD patients. While some studies support its use, particularly when patients start therapy with apomorphine, it is important to consider the risk of QT prolongation associated with domperidone [56]. Below, in Table 2, the pharmacological treatment in NOH is described.

In summary, pharmacological treatments play an essential role in managing orthostatic hypotension, helping to stabilize blood pressure and alleviate symptoms. However, careful monitoring is necessary to minimize potential side effects, such as supine hypertension and cardiovascular complications. These treatments should be part of a comprehensive approach that also includes patient education and non-pharmacological strategies to optimize outcomes and improve the overall quality of life for PD patients experiencing OH.

**Figure 1:**
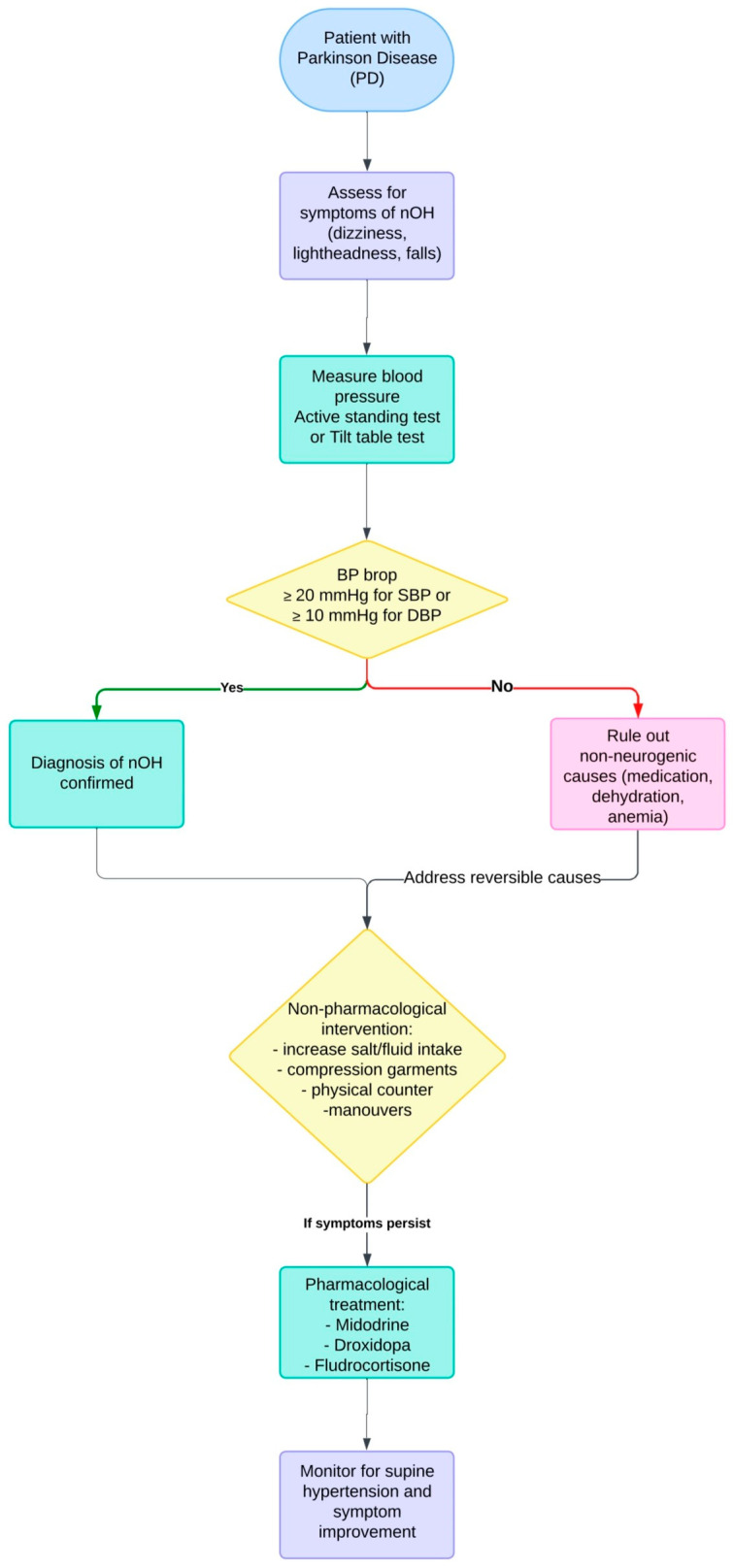
Management and treatment of NOH in PD.

Figure 1 provides a comprehensive overview of the management and treatment strategies for NOH in PD, highlighting key approaches and interventions tailored to patient needs.

One of the unresolved controversies in managing NOH in PD revolves around the significant variability in patient responses to available treatments. Pharmacological therapies, including midodrine, droxidopa, and fludrocortisone, are commonly prescribed to manage NOH by increasing vascular tone or expanding plasma volume. However, individual patient responses to these medications are often inconsistent, with some patients experiencing substantial symptom relief and others reporting limited efficacy or adverse effects. This variability may be influenced by disease stage, coexisting autonomic dysfunctions, medication interactions (particularly with dopaminergic agents), and the presence of comorbid conditions such as supine hypertension, which complicates treatment selection and dosing [19,57]. Moreover, the lack of large-scale, comparative clinical trials has led to uncertainty about which treatment strategies are most effective for specific patient subgroups, leaving clinicians to rely on trial-and-error approaches.

Another important issue is the management of the delicate balance between treating NOH and avoiding the exacerbation of supine hypertension, a frequent comorbidity in PD patients with autonomic dysfunction. Treatment strategies which are elaborated for elevating standing BP can sometimes worsen supine hypertension, increasing the risk of cardiovascular complications during rest or sleep. This dual burden complicates clinical decision-making, as there is no clear consensus on how best to manage both conditions simultaneously without compromising safety. Non-pharmacological interventions, such as physical counter-maneuvers, compression garments, and dietary modifications, have been suggested as complementary strategies, but their efficacy varies and is often not well-standardized in clinical practice [14,58]. Addressing these controversies requires more personalized approaches, better biomarkers for treatment response prediction, and comprehensive clinical trials to refine management protocols tailored to the complex autonomic profile of PD patients.

## 7. Other Abnormal BP Patterns in PD and PD-Plus Patients

NOH in PD patients frequently coexists alongside other abnormal BP patterns, including supine hypertension, nocturnal hypertension, and non-dipping hypertension. Non-sleeping hypertension (nSH) is defined as systolic BP ≥ 140 mmHg and/or diastolic BP ≥ 90 mmHg, measured after at least 5 min of rest in a supine position [10]. Remarkably, nearly 50% of patients with NOH also present with nSH. Although nSH is typically asymptomatic, it is associated with a higher risk of cardiovascular and cerebrovascular events. Supine hypertension can lead to nocturnal pressure natriuresis, which causes volume depletion and worsens NOH symptoms in the morning. Additionally, because nSH and NOH are hemodynamically opposites, the management of these conditions is a challenge for the clinician. Drugs that are used for NOH may have a pressure effect that can increase the risk of nSH. Non-pharmacological strategies for managing nSH include elevating the head of the bed to approximately 30° and consuming a carbohydrate-rich snack before bedtime to enhance blood flow to the splanchnic circulation. The patient should be given short-acting antihypertensive agents such as losartan, eplerenone, or captopril [59].

OH can also occur with postprandial hypotension (PPH) in PD patients. PPH, which is defined as a systolic BP drop of more than 20 mmHg within 2 h of food intake, is often underdiagnosed and represents an independent predictor of mortality. Regarding pathophysiology, the increased splanchnic blood flow that occurs after consuming a meal, reduces venous return and hence cardiac output. In PD patients with sympathetic dysfunction, vascular resistance does not increase accordingly in order to counteract reduced cardiac output. The most efficient non-pharmacological measures for PPH are consuming small quantitative meals more frequently and having water intake before meals [60].

NOH presents distinct patterns that can aid in differentiating PD from atypical Parkinsonian syndromes, including Parkinson-plus disorders such as MSA and progressive supranuclear palsy (PSP). In PD, NOH typically develops later in the disease course and is often milder due to the gradual involvement of autonomic pathways, as for MSA, where NOH can appear early and be more severe due to the widespread degeneration of autonomic centers in the brainstem and spinal cord. If the manifestation of NOH is in the early stages of MSA and is especially associated with urinary incontinence and cerebellar dysfunction, it strongly suggests an atypical Parkinsonian syndrome rather than idiopathic PD [38,61]. When distinguishing PD from Parkinson-plus syndromes with features of frontotemporal dementia (FTD), such as corticobasal degeneration (CBD) or PSP, the pattern of NOH can also be informative. These pathologies usually show less prominent autonomic dysfunction compared to MSA. However, cognitive and behavioral changes predominate in PD with FTD, while autonomic failure, including NOH, remains subtle or absent in the early stages. This is why for clinicians, a combination of marked NOH with minimal cognitive impairment would lean toward a diagnosis of MSA, whereas significant cognitive and behavioral disturbances with mild or absent NOH would be more indicative of PD with FTD features [62].

NOH also plays an important role in the transition from the early motor stages of PD to the late stages of dementia in PD, potentially serving as both a marker and contributor to cognitive decline. New evidence states that autonomic dysfunction, particularly NOH, reflects a larger process of neurodegeneration implicating brain regions involved in cognition, such as the cerebral cortex and limbic system. Studies have indicated that PD patients with NOH are at a higher risk of developing cognitive impairment and subsequent dementia, the underlying mechanisms involving chronic cerebral hypoperfusion due to repeated episodes of hypotension, leading to microvascular damage, white matter changes, and impaired neuronal function. This vascular burden may accelerate neurodegenerative processes, compounding the risk of cognitive decline in PD patients [63]. Clinically, the presence of NOH in PD patients could serve as a prognostic indicator for the progression to dementia related to PD, underscoring the need for early detection and management of autonomic dysfunction. Recognizing NOH in the course of PD may prompt clinicians to implement strategies aimed at stabilizing blood pressure to mitigate cerebral hypoperfusion and its cognitive consequences. This could involve pharmacologic treatments, lifestyle adjustments, and non-pharmacologic interventions to reduce orthostatic drops in blood pressure. Additionally, monitoring NOH might help identify patients at higher risk for dementia, allowing for early cognitive assessments and timely interventions. Addressing NOH could not only improve patients’ quality of life by reducing falls and syncope but also potentially slow the progression of cognitive decline in PD [64].

## 8. Discussions

Diagnosing NOH in PD remains complex due to overlapping symptoms with other autonomic dysfunctions found in these patients. Traditional diagnostic tools like the active standing test and tilt-table testing are valuable but may not always capture intermittent hypotensive episodes, especially in the early stages of PD. Recent advances in this area, such as the ΔHr/ΔSBP ratio, have demonstrated promising sensitivity and specificity in distinguishing NOH from non-neurogenic forms of OH. Still, variations in diagnostic performance across studies emphasize the need for further validation and standardization. Early and better detection of NOH is very important, not only to prevent complications like falls and syncope but also to detect patients at higher risk for cognitive decline and dementia, pointing out the necessity of thorough autonomic function testing in the routine management of PD.

The management of NOH in PD is particularly challenging due to the delicate balance between treating OH and avoiding exacerbation of coexisting supine hypertension. Non-pharmacological strategies, such as increased fluid and salt intake, physical counter-maneuvers, and the use of compression garments, serve as first-line interventions. Pharmacological treatments, including droxidopa and midodrine, offer symptomatic relief by enhancing vascular tone and increasing blood volume. However, these therapies carry risks, such as supine hypertension and other cardiovascular complications, necessitating careful dosing and monitoring. New therapies, like NET inhibitors, are under investigation to provide more targeted treatment options. Despite these advancements, the significant variability in patient responses still remains, and therefore the need for personalized treatment approaches that take into consideration the disease stage, comorbidities, and potential drug interactions.

Recent studies have suggested a potential role for NOH in accelerating the progression of PD to Parkinson’s disease dementia (PDD), through chronic cerebral hypoperfusion caused by repeated episodes of hypotension, microvascular strokes, white matter changes, and impaired neuronal function. Identifying NOH as an early marker of widespread neurodegeneration may provide management strategies to stabilize BP and preserve cognitive function. Interventions to mitigate orthostatic drops in BP may not only improve immediate symptoms but also potentially slow the development of cognitive decline. This perspective stresses the need for integrated care models that combine autonomic monitoring with cognitive assessments to optimize outcomes for PD patients at risk of developing dementia.

## 9. Conclusions

NOH significantly impacts patients with PD, causing disability, increasing the risk of falls, reducing quality of life, and leading to higher healthcare expenses. Thus, early diagnosis and appropriate management are crucial. The ΔHr/ΔSBP ratio can be a useful tool for identifying NOH, but more research is needed to better understand the connection between dopaminergic medication and NOH. Once diagnosed, treatment should focus on improving symptoms and quality of life through a combination of non-pharmacological and pharmacological strategies, rather than solely aiming to normalize blood pressure. Although considerable progress has been made with the FDA approval of droxidopa and midodrine for NOH treatment and ongoing clinical trials of new medications like NET inhibitors, further studies are essential to fully comprehend the pathophysiology of NOH. This knowledge will be vital for developing personalized treatments that address both central and peripheric mechanisms of NOH.

## Figures and Tables

**Table 1:** Comparative table of diagnostic tests for NOH in PD.

Diagnostic Test	Sensitivity (%)	Specificity (%)	Practical Application in PD
Active Standing Test	70	85	Simple and quick, used in clinical settings to detect BP drop upon standing.
Tilt Table Test	80	90	More sensitive for detecting orthostatic hypotension, useful when symptoms are intermittent.
24 h Ambulatory Blood Pressure Monitoring (ABPM)	60	70	Monitors blood pressure fluctuations throughout the day, identifying episodic hypotension.
Valsalva Maneuver	75	80	Assesses autonomic reflexes but may be difficult in patients with severe motor symptoms.
Head-Up Tilt Test with Continuous BP Monitoring	85	88	Highly sensitive and specific, continuous monitoring allows detailed analysis of BP changes.

**Table 2:** Pharmacological treatment in NOH.

Medication	Action Mechanism	Clinical Effects	Adverse Events	Suitable Patients
Droxidopa	Prodrug converted to norepinephrine; enhances vasoconstriction and cardiac output.	Improves standing BP, reduces dizziness and syncope.	Headache, hypertension, nausea, fatigue.	Patients with NOH from PD, MSA, or LBD
Midodrine	Alpha-1 adrenergic agonist; increases peripheral vascular resistance and venous return.	Improves standing BP, reduces syncope episodes.	Supine hypertension, piloerection, pruritus, urinary retention.	Patients with severe NOH and preserved renal function.
Fludrocortisone	Synthetic mineralocorticoid; increases sodium retention and plasma volume, enhancing BP.	Improves blood volume and BP stability.	Hypokalemia, edema, hypertension, heart failure, weight gain.	Patients with low blood volume contributing to NOH symptoms.
Pyridostigmine	Acetylcholinesterase inhibitor; enhances parasympathetic tone and baroreflex sensitivity.	Reduces orthostatic symptoms and supports BP stabilization.	Diarrhea, abdominal cramping, increased salivation, nausea.	Patients with mild NOH or as adjunct therapy.
NET Inhibitors	Block norepinephrine transporter (e.g., atomoxetine, reboxetine); increase norepinephrine levels.	Enhances vasoconstriction and improves orthostatic BP.	Insomnia, increased heart rate, anxiety, dry mouth, hypertension.	Patients with NOH secondary to autonomic dysfunction.
Domperidone	Dopamine D2 receptor antagonist; improves gastric motility and mitigates postprandial hypotension.	Reduces postprandial hypotension and nausea.	QT prolongation, arrhythmias, headache, dizziness.	Patients with NOH experiencing significant postprandial symptoms.

## Data Availability

Not applicable.
